# Leonurine Inhibits Hepatic Lipid Synthesis to Ameliorate NAFLD via the ADRA1a/AMPK/SCD1 Axis

**DOI:** 10.3390/ijms251910855

**Published:** 2024-10-09

**Authors:** Wen Fan, Maoxing Pan, Chuiyang Zheng, Haiyan Shen, Dajin Pi, Qingliang Song, Zheng Liang, Jianwei Zhen, Jinyue Pan, Lianghao Liu, Qinhe Yang, Yupei Zhang

**Affiliations:** School of Traditional Chinese Medicine, Jinan University, Guangzhou 510632, China; fanww1999@stu2022.jnu.edu.cn (W.F.); maoxingpan@stu2018.jnu.edu.cn (M.P.); chuiyangz@stu2018.jnu.edu.cn (C.Z.); shy98@stu2022.jnu.edu.cn (H.S.); pdj9642@stu2019.jnu.edu.cn (D.P.); sql1207@stu2020.jnu.edu.cn (Q.S.); liangzheng@stu2021.jnu.edu.cn (Z.L.); zjw1999@stu2022.jnu.edu.cn (J.Z.); pjyflww@stu.jnu.edu.cn (J.P.); ht0924@stu2023.jnu.edu.cn (L.L.)

**Keywords:** leonurine, nonalcoholic fatty liver disease, ADRA1a/AMPK/SCD1 axis, lipid metabolism, transcriptomic, lipidomic

## Abstract

Leonurine is a natural product unique to the Lamiaceae plant *Leonurus japonicus Houtt.*, and it has attracted attention due to its anti-oxidative stress, anti-apoptosis, anti-fibrosis, and metabolic regulation properties. Also, it plays an important role in the prevention and treatment of nonalcoholic fatty liver disease (NAFLD) through a variety of biological mechanisms, but its mechanism of action remains to be elucidated. Therefore, this study aims to preliminarily explore the mechanisms of action of leonurine in NAFLD. Mice were randomly divided into four groups: the normal control (NC) group, the Model (M) group, the leonurine treatment (LH) group, and the fenofibrate treatment (FB) group. The NAFLD model was induced by a high-fat high-sugar diet (HFHSD) for 12 weeks, and liver pathological changes and biochemical indices were observed after 12 weeks. Transcriptomic analysis results indicated that leonurine intervention reversed the high-fat high-sugar diet-induced changes in lipid metabolism-related genes such as stearoyl-CoA desaturase 1 (*Scd1*), Spermine Synthase (*Sms*), AP-1 Transcription Factor Subunit (*Fos*), Oxysterol Binding Protein Like 5 (*Osbpl5*), and FK506 binding protein 5 (*Fkbp5*) in liver tissues. Kyoto Encyclopedia of Genes and Genomes (KEGG) enrichment analysis results suggest that leonurine may exert its lipid-lowering effects through the AMP-activated protein kinase (AMPK) signaling pathway. Liver lipidomic analysis showed that leonurine could alter the abundance of lipid molecules related to fatty acyl (FAs) and glycerophospholipids (GPs) such as TxB3, carnitine C12-OH, carnitine C18:1-OH, and LPC (20:3/0:0). Molecular biology experiments and molecular docking techniques verified that leonurine might improve hepatic lipid metabolism through the alpha-1A adrenergic receptor (ADRA1a)/AMPK/SCD1 axis. In summary, the present study explored the mechanism by which leonurine ameliorated NAFLD by inhibiting hepatic lipid synthesis via the ADRA1a/AMPK/SCD1 axis.

## 1. Introduction

Nonalcoholic fatty liver disease (NAFLD) is characterized by progressive pathological changes such as fatty hyperplasia, steatosis, lobular inflammation, and hepatocyte ballooning, sometimes accompanied by sinusoidal fibrosis [1]. Recent systematic evaluations and meta-analyses have found a 50.4% surge in prevalence over the past three years, affecting about 38% of the worldwide population [2]. Against swift urbanization, NAFLD is evolving into a global pandemic. It is a complex multifactorial disease whose exact etiology is not fully understood, and recent studies have suggested that its progressive development is associated with lipid accumulation, oxidative stress, endoplasmic reticulum stress, and lipotoxicity [3]. Despite significant advancements in understanding the pathogenesis of NAFLD in recent years, lifestyle interventions remain the primary recommended strategy for its management [4]. Researchers are actively exploring complementary and alternative therapeutic approaches. For instance, the novel drug Rezdiffra, a partial agonist of the thyroid hormone receptor (THR), has been shown to selectively activate THR in the liver, effectively reducing lipid accumulation, and has been approved by the Food and Drug Administration (FDA) for the treatment of NASH. Additionally, herbal therapies have demonstrated unique potential in the prevention and treatment of NAFLD, offering new possibilities for pharmacological interventions.

**Figure 1:**
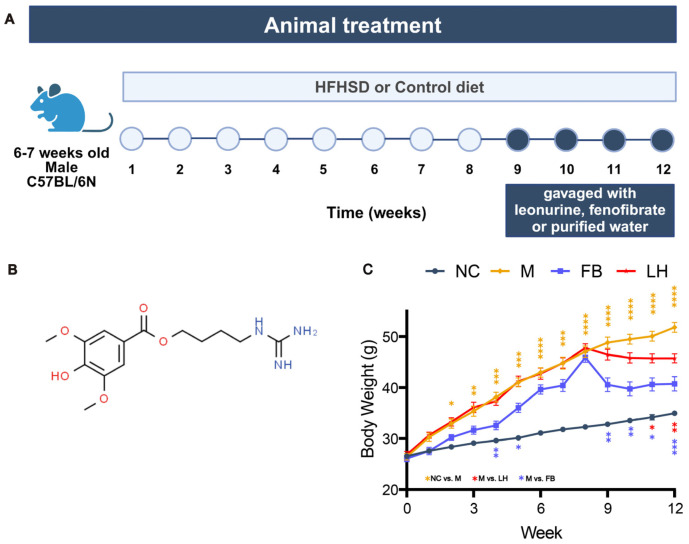
Figure of animal treatment and body weight. (**A**) The workflow of animal treatment. (**B**) Structural formula (C_15_H_18_N_2_O_9_) of leonurine. (**C**) Body weight. Data are presented as mean ± SEM (*n* = 10). * *p* < 0.05, ** *p* < 0.01, *** *p* < 0.001, **** *p* < 0.0001.

*Leonurus japonicus Houtt.*, commonly known as *Motherwort*, is a traditional medicinal herb in the Lamiaceae family. It has been widely used in gynecological treatments for over two thousand years across Asia and Europe [5,6]. As one of the principal bioactive components of Motherwort, leonurine is a unique natural product of the Leonurus genus (Figure 1B) [7]. According to *Pharmacopoeia of the People’s Republic of China* (2020), leonurine serves as the primary official indicator for monitoring the quality of Motherwort and its preparations. In recent years, with the rising incidences of NAFLD worldwide, leonurine has garnered significant attention for its antioxidative stress [8], anti-apoptotic [9], antifibrotic [10], and metabolic regulatory properties [11]. The lipid-lowering effect of leonurine and its safety have been validated in various animal models, including mice, rabbits, rhesus monkeys, etc. [12]. However, its specific mechanism against NAFLD remains unclear.

With the advancement and refinement of high-throughput technologies, various omics approaches have been increasingly applied in scientific research, providing valuable insights into mechanisms and accelerating the discovery of new drug targets. Therefore, in this study, we utilized lipidomics and transcriptomics to investigate the effects of leonurine on a high-fat and high-sugar diet (HFHSD)-induced NAFLD mice model and to explore its potential mechanisms. We hoped that this research would provide a deeper understanding of the mechanisms about how leonurine improves NAFLD, thereby offering an experimental basis for NAFLD treatment.

## 2. Results

### 2.1. Four Weeks of Leonurine Treatment Resulted in Significant Weight Loss in HFHSD-Induced Mice

We investigated the effects of leonurine on a mouse model of NAFLD induced by HFHSD for 12 weeks. During the establishment of the model, the NAFLD mice were administered leonurine orally once daily for 4 weeks (Figure 1A). As depicted in Figure 1C, we monitored the dynamic changes in body weight of the mice in each group, focusing on comparisons between the NC and M groups, and between the M and LH groups. Data showed that from the second week of the experiment, the body weight of the mice in each group steadily increased. From the third week, the weight difference induced by HFHSD between the NC and M groups became statistically significant. The body weight curve of the two treated groups began to decline at the 8th week. After leonurine treatment, body weight decreased, and compared with the M group mice, the LH group significantly reversed the weight gain induced by HFHSD modeling.

### 2.2. Leonurine Improved Serum and Hepatic Biochemical Parameters in NAFLD Mice

**Figure 2:**
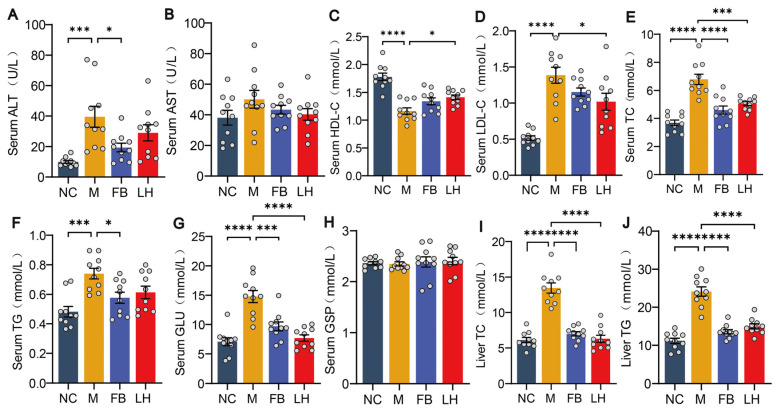
Biochemical parameters of mice. (**A**) Serum ALT. (**B**) Serum AST. (**C**) Serum HDL-C. (**D**) Serum LDL-C. (**E**) Serum TC. (**F**) Serum TG. (**G**) Serum GLU. (**H**) Serum GSP. (**I**) Liver TC. (**J**) Liver TG. Data are presented as mean ± SEM (*n* = 10). * *p* < 0.05, *** *p* < 0.001, **** *p* < 0.0001.

Due to the close relationship between NAFLD and glucose and lipid metabolism, we measured these biochemical parameters to assess the metabolic status and liver function of the mice. GLU reflects the overall metabolic status and insulin sensitivity of mice, while GSP is used to assess the blood glucose control of mice over the past few weeks, which is important for detecting the pathophysiological changes in NAFLD and insulin resistance. The results show that GLU and GSP levels increased in all groups compared to the NC group and decreased after drug treatment (Figure 2G–H). ALT and AST are markers of liver injury, with elevated levels usually associated with hepatocyte damage or inflammation. Compared to the NC group, all groups showed elevated ALT and AST levels; ALT significantly decreased after leonurine treatment, while AST decreased but not significantly (Figure 2A,B). We believe that a decrease in HDL-C or an increase in LDL-C may be associated with lipid metabolism disorders. As we speculated, after HFHSD induction, HDL-C levels decreased and LDL-C levels increased in all groups of mice. This trend was reversed after leonurine treatment (Figure 2C,D). Serum TC and TG levels reflect the overall lipid metabolism status of mice, while liver TC and TG directly reflect cholesterol levels and lipid accumulation in the liver and hepatocyte steatosis, closely related to the pathogenesis of NAFLD. The results demonstrated that the trends in liver TC and TG levels mirrored those in the serum (Figure 2I,J). Compared to the NC group, the M, LH, and FB groups showed elevated levels, which significantly decreased following leonurine treatment (Figure 2E,F). These data indicate that leonurine effectively mitigated the liver injury and lipid metabolism disorders induced by a high-fat diet in NAFLD mice, demonstrating its potential therapeutic benefits.

### 2.3. Leonurine Reversed Hepatic Histopathologic Changes in Mice with NAFLD

**Figure 3:**
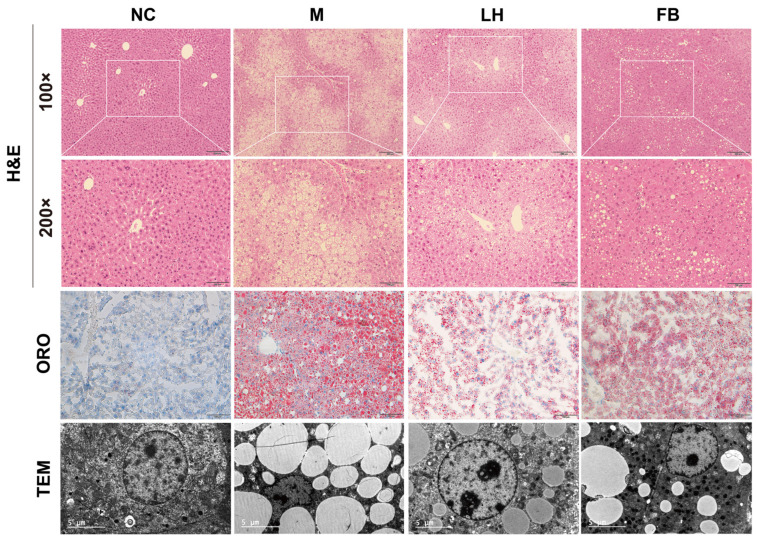
Pathological observation of mice. Representative images of H&E staining of liver paraffin sections, The white box is a ×200 microscope (100× and 200×), ORO staining of frozen liver slides (200×) observed under a microscope and liver ultrathin sections observed under TEM (5800×).

Pathology is the gold standard for diagnosing NAFLD. We performed H&E staining, ORO staining, and TEM observation to comprehensively observe the pathological changes in liver tissue. As shown in Figure 3, H&E staining in all groups except the NC group revealed disordered hepatocyte arrangement, numerous small lipid droplets in the cytoplasm, and cell swelling. There was some improvement in the LH group compared to the M group. The observations from ORO-stained sections also supported this view. In the TEM micrographs of liver sections, the M group showed irregular nuclear morphology, with some nuclei having chromatin condensed into discrete clumps, along with numerous lipid droplets. Significant improvement was observed after leonurine treatment, further demonstrating its therapeutic potential in NAFLD.

### 2.4. Leonurine Regulated Hepatic RNA Expression Profile in NAFLD Mice

**Figure 4:**
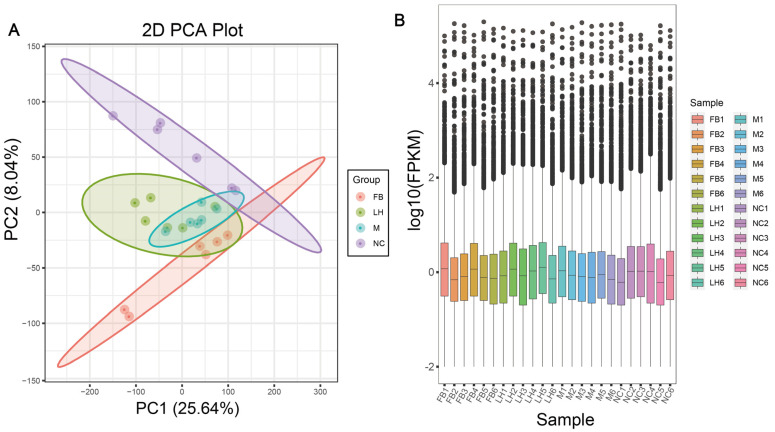
Sample analysis plots for transcriptomics. (**A**) PCA plot. (**B**) Expression box plot: The *x*-axis represents different samples, while the *y*-axis represents the logarithmic value of sample expression levels in FPKM.

To explore the potential mechanisms by which leonurine prevents NAFLD, we performed RNA sequencing analysis on liver samples. Initially, we evaluated the samples from each group, as shown in the principal component analysis (PCA) in Figure 4A, which indicated high intra-group clustering and good reproducibility, thus allowing for further analysis. We used fragments per kilobase of transcript per million fragments mapped (FPKM) as the metric for transcript or gene expression levels, with the FPKM calculation formula provided in the Supplementary Materials. Based on the FPKM values, we plotted expression box plots (Figure 4B), and the gene expression distribution of the samples in each group was concentrated, indicating that the differences in gene expression levels between the samples in each group were small. This consistency further verifies the credibility of the experimental results.

**Figure 5:**
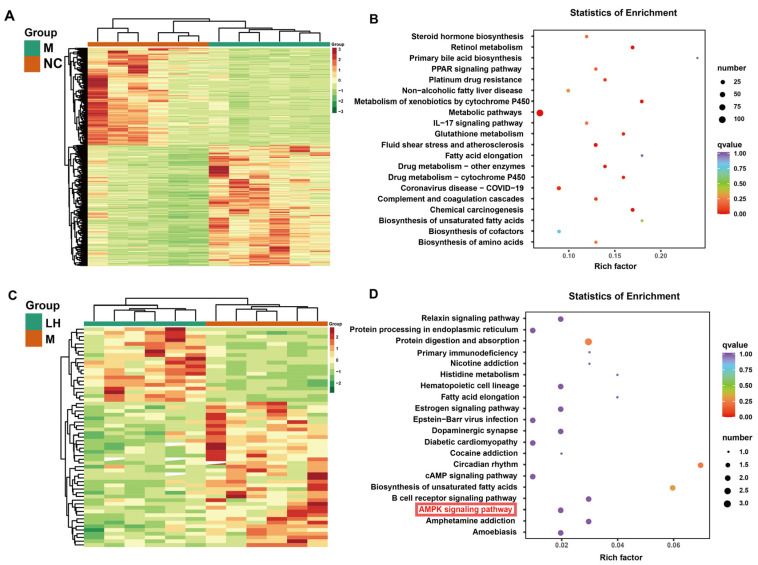
DEGs analysis for transcriptomics. (**A**) DEGs between the NC and M groups: The *x*-axis denotes sample names and hierarchical clustering results, while the *y*-axis represents DEGs and their hierarchical clustering results. Red indicates high expression, and green indicates low expression. (**B**) Enrichment dot plot comparing NC and M groups: The *y*-axis shows KEGG pathways, and the *x*-axis represents the Rich factor. A larger Rich factor indicates a higher degree of enrichment. Larger dots signify a greater number of DEGs enriched in the pathway, and redder dots indicate more significant enrichment. (**C**) Clustering heatmap of DEGs between the M and LH groups. (**D**) Enrichment dot plot comparing M and LH groups.

Subsequently, differentially expressed genes (DEGs) in each group were normalized and subjected to hierarchical clustering analysis, producing the heatmaps illustrated in Figure 5A,C. Comparing the NC and M groups revealed 826 DEGs, with 458 up-regulated and 368 down-regulated genes. Comparing the M and LH groups identified 59 DEGs, with 20 up-regulated and 39 down-regulated genes, as detailed in the Transcriptomics Supplementary Materials. Twenty key differential genes, including collagen alpha-1(III) (*Col3a1*), Spermine Synthase (*Sms*), AP-1 Transcription Factor Subunit (*Fos*), Oxysterol Binding Protein Like 5 (*Osbpl5*), and FK506 binding protein 5 (*Fkbp5*), were identified through comparative analyses of differential genes in the NC vs. M and M vs. LH groups, which can be found in the Transcriptomics Supplementary Materials. We conducted Kyoto Encyclopedia of Genes and Genomes (KEGG, https://www.genome.jp/kegg, accessed on 24 November 2021.) pathway analysis on the DEGs to preliminarily explore their functional mechanisms, presenting the results in bubble plots (Figure 5B,D). As shown in Figure 5B, DEGs in metabolic pathways and PPAR signaling pathways were enriched after ingestion of the HFHSD compared to the NC group. The results indicated that leonurine treatment of NAFLD is closely related to the cyclic adenosine monophosphate (cAMP) signaling pathway, protein digestion and absorption, and the AMPK signaling pathway. As a key regulator of liver metabolism, Peroxisome Proliferator Activated Receptor Alpha (PPARα) was activated in rodent models, thereby improving hepatic steatosis, inflammation, and fibrosis [13]. And the AMPK signaling pathway is closely associated with lipid metabolism [14]. These findings suggest that the protective effects of leonurine against NAFLD may be mediated through the modulation of these key metabolic pathways, offering potential therapeutic targets for the treatment and prevention of NAFLD.

### 2.5. Leonurine Modulates Hepatic Lipid Profiles in NAFLD Mice

**Figure 6:**
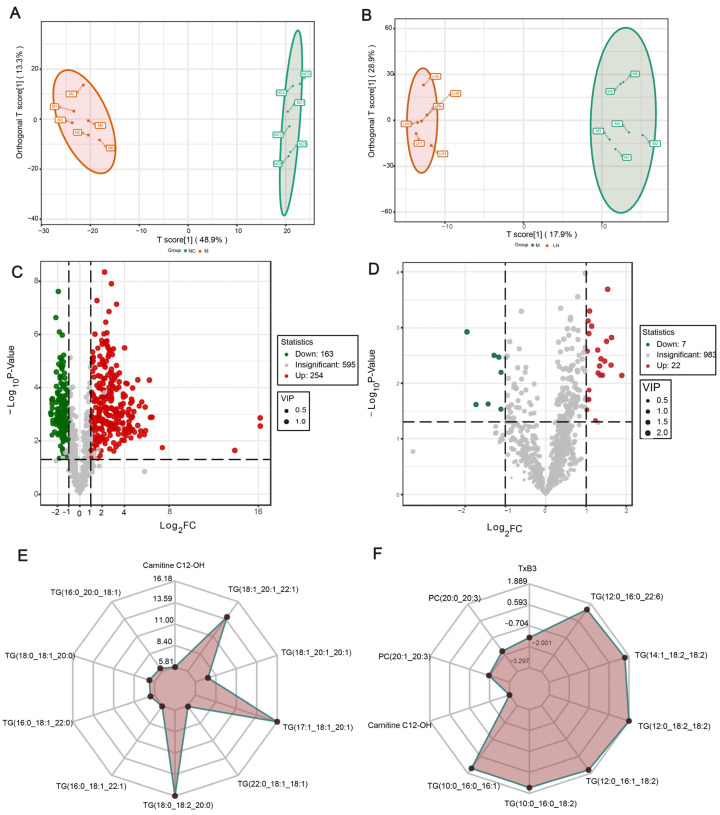
Lipidomics analysis results. (**A**) OPLS-DA score plot for NC group vs. M group: the *x*-axis represents the predicted component scores, showing inter-group differences; the *y*-axis represents the orthogonal component scores, showing intra-group differences; the percentages indicate the explained variance for each component. (**B**) OPLS-DA score plot for M group vs. LH group. (**C**) Volcano plot of differential lipid molecules for NC group vs. M group: each point represents a lipid molecule, with green points indicating down-regulated differential lipid molecules, red points indicating up-regulated differential lipid molecules, and gray points indicating lipid molecules detected but not significantly different. (**D**) Volcano plot of differential lipid molecules for M group vs. LH group. (**E**) Radar plot of differential lipid molecules for NC group vs. M group: the grid lines correspond to log_2_FC, the log_2_-transformed fold change in differential lipid molecules. The green shading is formed by connecting the log_2_FC values of each substance. (**F**) Radar plot of differential lipid molecules for M group vs. LH group.

**Figure 7:**
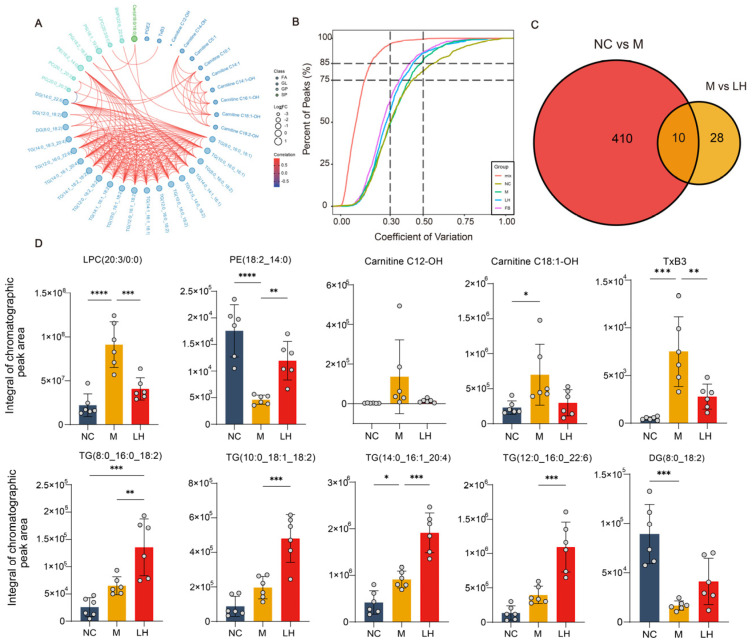
Critical analysis of lipid molecules. (**A**) Correlation analysis plot of differential lipid molecules for M group vs. LH group: The plot shows the Pearson correlations between different lipid molecules, with green lines indicating positive correlations and blue lines indicating negative correlations. The size of the points represents the log_2_FC values of the lipid molecules. (**B**) CV distribution plot for each group: The *x*-axis represents the CV values, and the *y*-axis represents the proportion of substances with CV values below the corresponding threshold in the total number of substances. Different colors represent different groups, with mix denoting QC samples. Two reference lines perpendicular to the *x*-axis correspond to CV values of 0.3 and 0.5, while two reference lines parallel to the *x*-axis correspond to 75% and 85% of the total number of substances. (**C**) Venn diagram. (**D**) Statistical plot of the 10 key differential lipid molecules for M group vs. LH group. Data are presented as mean ± SEM. * *p* < 0.05, ** *p* < 0.01, *** *p* < 0.001, **** *p* < 0.0001.

Given that leonurine has a regulatory effect on lipids, we conducted lipidomic analyses on liver samples from various groups of mice. Initially, we performed quality control (QC) analysis using the coefficient of variation (CV) values of the samples. The QC samples were prepared by mixing sample extracts to monitor the reproducibility of the analysis under the same treatment conditions. As shown in Figure 7B, more than 75% of the substances in the QC samples had CV values less than 0.3, indicating that the experimental data were very stable and suitable for further analysis. The integral of the chromatographic peak area can quantify the lipid content in samples, which is helpful to compare sample differences, analyze metabolic pathways, and ensure the quality control of the method, which is of great significance in lipidomic research. We then performed comparative lipidomic analyses of the M and NC groups and the M and LH groups. As depicted in the orthogonal partial least squares discriminant analysis (OPLS-DA) score plots in Figure 6A,B, the variability among and within the groups was minimal. We screened for different lipid molecules using the criteria of fold change ≥ 2, fold change ≤ 0.5, and variable importance in the projection (VIP) ≥ 1. As illustrated in the volcano plots in Figure 6C,D, compared to mice on a normal diet, there were 420 lipid molecules with significantly altered abundances in the livers of NAFLD mice induced by HFHSD, with 256 lipid molecules showing increased abundance and 163 showing decreased abundance. Following leonurine treatment, 38 lipid molecules were altered in NAFLD mice, including 15 down-regulated and 23 up-regulated, as detailed in the heatmap (Figure S1); the primary lipid classes involved are glycerolipids (GLs), fatty acyls (FAs), glycerophospholipids (GPs), and sphingolipids (SPs).

Next, we selected the top 10 lipids with the most significant differences to create a radar plot (Figure 6E,F). The differential lipid molecules between the NC group and M group include carnitine C12-OH, TG(16:0_20:0_18:1), TG(18:0_18:1_20:0), TG(16:0_18:1_22:0), TG(16:0_18:1_22:1), TG(18:0_18:2_20:0), TG(22:0_18:1_18:1), TG(17:1_18:1_20:1), TG(18:1_20:1_20:1), and TG(18:1_20:1_22:1). And the differential lipid molecules between the M group and LH group include TxB3, PC(20:0_20:3), PC(20:1_20:3), carnitine C12-OH, TG(10:0_16:0_16:1), TG(10:0_16:0_18:2), TG(12:0_16:1_18:2), TG(12:0_18:2_18:2), TG(14:1_18:2_18:2), and TG(12:0_16:0_22:6). Increased levels of carnitine C12-OH in the M group compared to the NC group suggest an alteration in fatty acid oxidation, a key process in energy metabolism. The reduction in carnitine C12-OH levels in the LH group after leonurine treatment indicates a potential normalization of fatty acid oxidation. The significant changes in various triglyceride species between the groups underscore the impact of NAFLD on lipid metabolism. Elevated levels of TG species such as TG(16:0_20:0_18:1), TG(18:0_18:1_20:0), and TG(18:1_20:1_22:1) in the M group highlight the hepatic accumulation of fats, a hallmark of NAFLD. The reduction in these TG species in the LH group suggests that leonurine may help reduce hepatic fat accumulation and improve lipid metabolism. The differential levels of PCs such as PC(20:0_20:3) and PC(20:1_20:3), along with TxB3, between the M and LH groups indicate changes in membrane lipid composition and inflammation, respectively. PCs are essential components of cell membranes [15], and their alteration may reflect changes in cell membrane integrity and function. TxB3, a marker of platelet activation and inflammation [16], being lower in the LH group suggests an anti-inflammatory effect of leonurine.

Considering the synergistic or antagonistic relationship between different lipid molecules, we further performed Pearson’s correlation analysis on the distinctly different lipid molecules screened from groups M and LH (Figure 7A). The results showed a negative correlation between DG(14:0_22:6) and PC(20:0_20:3), while other lipid molecules exhibited positive correlations. Finally, we performed an intersection analysis of differential lipid molecules between the M and NC groups, as well as between the M and LH groups, identifying 10 key differential lipid molecules. These lipid molecules included FAs, GPs, and GLs. Among them, the abundances of Thromboxane B3 (TxB3), carnitine C12-OH, carnitine C18:1-OH, and LPC(20:3/0:0) were reduced following LH (Figure 7C,D). These differences in lipid molecules between the groups provide insights into the biochemical pathways disrupted by NAFLD and the potential mechanisms by which leonurine exerts its therapeutic effects. Also, they highlight leonurine’s role in modulating fatty acid oxidation, reducing hepatic lipid accumulation, and exerting anti-inflammatory effects, which could contribute to its overall beneficial impact on NAFLD.

### 2.6. Leonurine Regulated Hepatic ADRA1a/AMPK/SCD1 Axis in HFHSD-Induced NAFLD Mice

**Figure 8:**
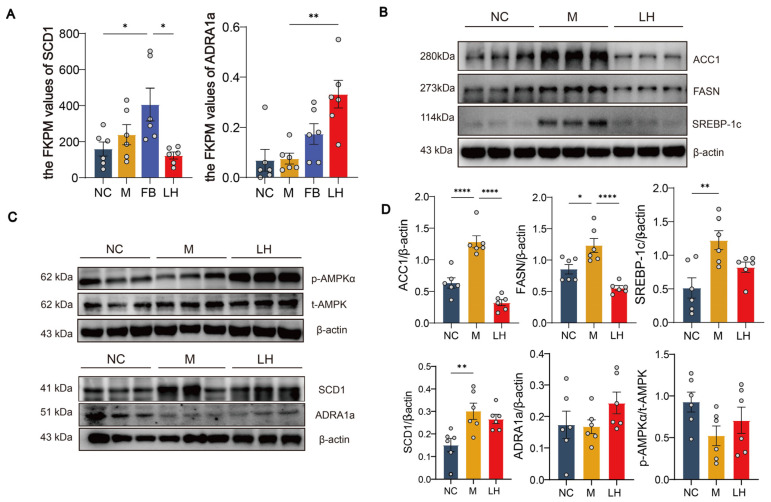
LH protects against NAFLD by activating ADRA1a/AMPK/SCD1 axis. (**A**) FPKM statistics for ADRA1a and SCD1 (*n* = 6). (**B**) Protein expression diagram of ACC1, FASN, and SREBP-1c, with β-actin as the internal reference (*n* = 6). (**C**) Protein expression diagram of p-AMPKα, t-AMPK, ADRA1a, and SCD1, with β-actin as the internal reference (*n* = 6). (**D**) Statistical maps of protein expression of SCD1/β-actin, ADRA1a/β-actin, p-AMPKα/t-AMPK, ACC1/β-actin, FASN/β-actin, and SREBP-1c/β-actin (*n* = 6). Data are presented as mean ± SEM. * *p* < 0.05, ** *p* < 0.01, **** *p* < 0.0001.

Considering the significant biological role of the AMPK signaling pathway in liver lipid metabolism, we preliminarily explored the regulatory effects of leonurine on the ADRA1a/AMPK/SCD1 signaling cascade in liver tissues of HFHSD-induced NAFLD mice. Transcriptomic results indicated that leonurine treatment led to a significant enrichment of the AMPK signaling pathway, with notable changes in the gene expression of its upstream protein ADRA1a and downstream protein SCD1 (Figure 8A). To further investigate, we measured the levels of SCD1, ADRA1a, t-AMPK, and p-AMPKα using WB analysis. The results revealed a significant increase in the p-AMPKα/t-AMPK ratio, indicating that leonurine treatment enhanced AMPK activation through phosphorylation (Figure 8C,D). Additionally, WB analysis of the downstream proteins involved in fatty acid synthesis within the AMPK signaling pathway—namely SREBP-1c, ACC1, and FASN—showed significant reductions in their levels (Figure 8B–D). This suggested that leonurine treatment reduced the expression of these key proteins involved in lipid synthesis. Moreover, the observed increase in ADRA1a protein expression implies that leonurine may enhance ADRA1a activity, potentially activating associated signaling pathways. Concurrently, the significant decrease in SCD1 expression points to reduced hepatic lipid synthesis and accumulation. Overall, these findings suggest that leonurine regulates the ADRA1a/AMPK/SCD1 signaling axis, effectively modulating hepatic lipid metabolism in NAFLD and inhibiting lipid synthesis.

### 2.7. Effects on Immunofluorescence

**Figure 9:**
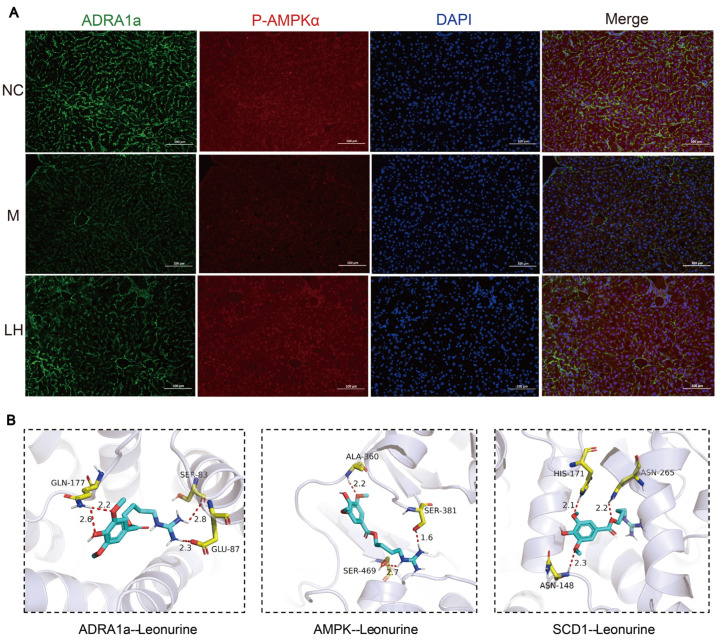
IF and molecular docking results. (**A**) IF assay of ADRA1a (green) and p-AMPKα (green) in the livers of mice in the NC, M, and LH groups, and DAPI was used for nucleus staining (blue). (100 μm, 200×). (**B**) Molecular docking: blue cartoon indicates the protein, yellow stick indicates the interacting amino acids, the cyan stick represents LH, the red dashed line represents the hydrogen bonding interaction, and the values on the dashed line represent the hydrogen bonding distance in micrometers.

To further elucidate the association between p-AMPKα and ADRA1a, we employed an immunofluorescence double-staining technique. The results demonstrated that both ADRA1a and p-AMPKα were localized in the cytoplasm and nucleus. In the context of NAFLD, AMPK activation occurs through phosphorylation, which plays a crucial role in responding to intracellular energy stress and mitochondrial damage. After leonurine treatment, the fluorescence intensity of both ADRA1a and p-AMPKα in liver tissues was significantly increased, indicating the elevated levels and activity of these proteins (Figure 9A). This enhancement suggests that leonurine may facilitate lipid degradation through AMPK phosphorylation. Consequently, leonurine likely regulates the expression of lipid synthesis-related proteins downstream of the ADRA1a/AMPK/SCD1 axis, thus contributing to improved hepatic lipid metabolism in HFHSD-induced NAFLD mice.

### 2.8. Molecular Docking of Leonurine with ADRA1a, AMPK, and SCD1 Proteins

Molecular docking technology is a convenient and effective method for studying the interactions between small molecules and their target proteins. To preliminarily assess the binding ability and interaction modes of leonurine with the ADRA1a/AMPK/SCD1 axis, we conducted docking studies using Vina 1.1.2 software. The interaction maps for leonurine with ADRA1a, AMPK, and SCD1 proteins, as shown in Figure 9B, illustrate the binding sites and modes of action of leonurine on these proteins. On the ADRA1a protein, leonurine formed four hydrogen bonds with residues GLU-87, GLN-177, and SER-83, with bond lengths of 2.3 Å, 2.8 Å, 2.2 Å, and 2.6 Å, respectively. With AMPK, leonurine established three hydrogen bonds with the residues ALA-360, SER-381, and SER-469, showing bond lengths of 2.2 Å, 1.6 Å, and 2.7 Å, respectively. For the SCD1 protein, leonurine formed three hydrogen bonds with residues ASN-148, HIS-171, and ASN-265, with bond lengths of 2.1 Å, 2.2 Å, and 2.3 Å, respectively. These interactions suggest that these amino acids are crucial for binding. Negative values for binding affinity indicate possible binding, with smaller values reflecting a higher likelihood of binding. According to the scores provided by Vina 1.1.2 software, as shown in Table 1, leonurine demonstrates binding potential with all three proteins, with SCD1 exhibiting the highest binding affinity, followed by ADRA1a and AMPK.

## 3. Discussion

In this study, we preliminarily explored the mechanism of leonurine in NAFLD using lipidomics and transcriptomics techniques. The results indicate that leonurine may exert its lipid-lowering effects through the ADRA1a/AMPK/SCD1 axis. This study provides important insights into the therapeutic mechanisms of NAFLD.

NAFLD is a persistent disease associated with obesity, type 2 diabetes, insulin resistance, and hyperlipidemia. It is characterized by the accumulation of lipids in the liver, which can lead to cirrhosis and hepatocellular carcinoma [17]. The evolution of NAFLD reflects a diversity of environmental factors, microbiota, metabolism, comorbidities, and genetic risk factors [18]. Leonurine, a natural small molecule compound, has garnered significant attention for its remarkable anti-inflammatory and lipid-lowering effects. Studies have shown that leonurine inhibits LPS-induced inflammation via the TLR4-mediated NF-κB pathway, reducing the expression levels of TNF-α and IL-1β [19]. Its metabolism mainly involves four pathways: glycerophospholopid metabolism, linoleic acid metabolism, tryptophan metabolism, and glutamate metabolism [20]. Song et al. combined leonurine with nanotechnology, demonstrating favorable in vivo drug release kinetics, significantly improving lipid levels, and reducing liver damage, showcasing promising clinical translation potential [21]. Previous studies have shown that the dosage of 30 mg/kg/d leonurine is widely used based on its proven effectiveness and safety in disease treatment, so we used this dose of an in-depth study [22,23].

The HFHSD is commonly employed to induce animal models of NAFLD, recapitulating key pathogenic and histological features observed in human NAFLD [24]. Therefore, in the present study, we utilized the HFHSD to induce a mouse model of NAFLD. By evaluating the basal indices, biochemical indices, and pathological changes, we found that HFHSD rapidly increased the body weight of mice and dysregulated serum and liver indices related to glucose and lipid metabolism, and that leonurine treatment reduced body weight and ameliorated hepatic and systemic lipid abnormalities in NAFLD mice.

The results of the transcriptomic study provide important clues for exploring the anti-NAFLD mechanism of leonurine. Transcriptome sequencing showed that 20 genes, including Col3a1, Sms, Fos, Osbpl5, and Fkbp5, were differentially expressed between the M and LH groups and between the NC and M groups. Col3a1 is usually closely associated with the fibrotic process in NAFLD [25]. Expression of the Fkbp5 gene is associated with reduced glucose uptake and acts as a negative regulator of the protein kinase B (PKB) pathway, leading to impaired insulin signaling, which is expected to prevent high-fat diet-induced obesity [26,27]. Additionally, Osbpl5 inhibits cholesterol transport, and Sms effectively reduces triglyceride storage [28,29]. Alterations in these genes may affect the expression of key lipogenic genes or proteins in the liver although, of course, the molecular mechanisms by which leonurine regulates these genes require further experimental confirmation. Of interest, the AMPK signaling pathway, which is closely related to lipid synthesis, was significantly enriched in the transcriptome KEGG analysis, ADRA1a was down-regulated in the NAFLD environment, but significantly increased after treatment with leonurine, and SCD1 was the opposite. We explored this pathway.

ADRA1a, a member of the G protein-coupled receptor family, is widely expressed on cell surfaces and within cells, stimulating the sympathetic nervous system (SNS) through binding with catecholamines [30]. Previous studies have shown that its activation can induce hepatic glycogenolysis and gluconeogenesis [31,32]. The use of ADRA1a antagonists can block SNS, enhance hepatic progenitor cell accumulation, and alleviate hepatic necrosis and steatosis [33]. Under low-energy conditions, AMPK phosphorylates specific enzymes and growth control nodes to increase ATP production and decrease ATP consumption, acting as a cellular energy sensor involved in maintaining energy balance in various tissues [34]. As a key upstream molecule of AMPK, ADRA1a can directly activate AMPK and regulate its downstream proteins [35]. The activation of the AMPK signaling pathway relies on phosphorylation, particularly the activation of p-AMPKα (Thr172), which reduces lipid synthesis by modulating enzyme activity. In past studies, ADRA1a activation was found to cause p-AMPKα activation [36], thereby influencing lipid synthesis and glucose homeostasis [34,37,38]. Our results showed that the expression of p-AMPKα was reduced in group M mice, suggesting that chronic intake of the HFHSD decreases hepatic p-AMPKα expression. In contrast, leonurine treatment increased p-AMPKα expression. The increase in the content and activity of ADRA1a and p-AMPKα in cells further confirmed this view (Figure 8A). Taken together, the expression of p-AMPKα was enhanced after leonurine treatment, indicating that the regulation of ADRA1a plays a crucial role in improving NAFLD. SCD1, a rate-limiting enzyme in lipid synthesis, preferentially catalyzes the biosynthesis of monounsaturated fatty acids from palmitoyl-CoA and stearoyl-CoA, forming palmitoleoyl-CoA and oleoyl-CoA, respectively. These monounsaturated fatty acids are key components of membrane phospholipids [39,40,41]. Past studies have found that SCD1 can help in the organization of different organisms in the accumulation of FAs [42,43,44]. AMPK phosphorylation inhibited SCD1 expression and showed synergistic expression with changes in FA content [45]. As mentioned above, lipidomics results showed a significant reduction in the levels of GPs and FAs after leonurine treatment. We preliminarily speculated that leonurine promotes the expression of ADRA1a, phosphorylates and activates the AMPK signaling pathway, decreases the expression of SCD1, and decreases the levels of GPs and FAs in liver, thereby exerting its anti-NAFLD effect.

In lipidomics analysis, correlation analysis using significantly different lipid molecules (Figure 7A) reveals a negative correlation between DG(14:0_22:6) and PC(20:0_20:3). Both are glycerophospholipids regulated by specific enzyme-catalyzed reactions: PC is hydrolyzed by phospholipase C (PLC) to produce DG and phosphatidylcholine, while DG is converted back to PC via the CDP–choline pathway. This interconversion is essential not only for cellular signaling but also for maintaining the stability of cell membrane structure and function [46,47]. Hence, the negative correlation between DG(14:0_22:6) and PC(20:0_20:3) may stem from enzyme-catalyzed reactions and regulatory feedback mechanisms. However, the mechanism of negative feedback between lipid molecules requires further deeper investigation. After treatment with leonurine, compared with lipid molecules in the M group, it was suggested that leonurine could significantly improve FA-related lipid molecules including TxB3, Prostaglandin E2 (PEG2), Camitine C12-0H, Camitine C14-0H, Camitine C5:1, Camitine C10:1, and so on. Meanwhile, some GPs’ contents were reversed, including PC(20:0_20:3), PC(20:1_20.3), LPC (20:3/0:0), and BMP(22:6_22:6) (Figure S1). Subsequently, we treated the differential lipid molecules between the NC and M groups, and between the M and LH groups, and we identified 10 key lipid molecules including GPs (such as PE(18:2_14:0) and LPC(20:3/0:0)), triglycerides (such as TG(8:0_16:0_18:2), TG(10:0_18:1_18:2), TG(12:0_16:0_22:6)), diacylglycerols (such as DG(8:0_18:2)), and others like TxB3 and carnitine derivatives. Interestingly, the increase in certain triglyceride levels suggests a complex regulation of lipid metabolism pathways by leonurine. In the liver, glycerol-3-phosphate undergoes a series of acylation reactions to form TG, with diacylglycerol acyltransferase (DGAT) being the key enzyme in this pathway [48]. Specifically, the increased activity of DGAT2 significantly promotes TG synthesis in the liver, with some TG being secreted in the form of very-low-density lipoprotein (VLDL) [49]. Therefore, we suggest that this may be related to the enhanced activity of key enzymes in the Kennedy pathway or an increased supply of the substrate glycero-3-phosphate [50]; however, further investigation is needed.

SREBP-1c, ACC1, FASN, and SCD1 are triglyceride synthesis-related proteins, regulated by the AMPK signaling pathway [51,52]. The WB results show that their protein expression levels were all reduced after leonurine treatment [53,54] (Figure 7B). It was further confirmed that leonurine affected lipid anabolism in NAFLD mice by participating in AMPK signaling pathway. At last, to further verify the involvement of the ADRA1a/AMPK/SCD1 axis, we used molecular docking technology. A negative value for binding affinity indicates the possibility of binding, and generally a smaller number is considered to be more likely to bind. In this study, we performed three dockings in parallel, and the docking software gave the average binding affinity values of leonurine with ADRA1a, AMPK, and SCD1 as −6.716, −6.295, −8.491. We can see that leonurine has the potential to bind to all three proteins, which have binding potential. Among them, the binding effect of SCD1 is the best, followed by ADRA1a and AMPK.

Taken together, our study suggests that leonurine may reduce the accumulation of lipids such as GPs and FAs in HFHSD-induced NAFLD mice, possibly by activating the ADRA1a/AMPK/SCD1 axis through the phosphorylation of AMPK protein. These findings provide new evidence for the clinical translation of leonurine. However, this study has limitations. In future studies, we will further elucidate the interaction between ADRA1a and SCD1 using more molecular biology experiments, further validate the ADRA1a/AMPK/SCD1 axis by using in vitro experiments, and trace the dynamic changes in lipids in living cells using fluorescence labeling and microscopic observation, in order to fully explain the mechanism of action of leonurine against NAFLD.

## 4. Materials and Methods

### 4.1. Reagents and Antibodies

Leonurine hydrochloride (CAS No. 24735-18-0, Cat. Y-202-1g, purity > 98%) was purchased from Chengdu Herbpurify Biotechnology Co (Chengdu, China). Fenofibrate (Batch No.: 32670, Import Drug Registration Certificate No.: H20181239, Hong Kang, China.) was purchased from Recipharm fontaine. Primary antibodies included sterol-regulatory element binding protein-1c (SREBP-1c, Santa Cruz Biotechnology, Shanghai, China Cat. sc-365513), total AMP-activated protein kinaseα (t-AMPK, Cell Signaling Technology, Danvers, MA, USA, Cat. No. 2532), phospho-AMPKα (p-AMPKα, Cell Signaling Technology, MA, USA, Cat. No. 2535), fatty acid synthase (FASN, Cell Signaling Technology, MA, USA, Cat. No. 3180s), acetyl-CoA carboxylase 1 (ACC1, Cell Signaling Technology, MA, USA, Cat. No. 3676s), alpha-1A adrenergic receptor (ADRA1a, Beijing, China, Cat. #bs-0600R), stearoyl-CoA desaturase 1 (SCD1, Beijing Biosynthesis Biotechnology, Beijing, China, Cat. #bs-3787R), and β-actin (Cell Signaling Technology, MA, USA, Cat. No. 3700). Secondary antibodies were anti-rabbit IgG, horseradish peroxidase (HRP)-linked antibody (Cell Signaling Technology, MA, USA, Cat. No. 7074s), and anti-mouse IgG, HRP-linked antibody (Abbkine, Wuhan, China, Cat. No. A21010).

### 4.2. Animal Experiment

Forty SPF-grade male C57BL/6N mice (6–7 weeks old) were purchased from Beijing Vital River Laboratory Animal Technology Co., Ltd (Beijing, China). During the experiments, the mice were individually housed under standard conditions of 22 °C ± 2 °C, 55% ± 5% humidity, and a light–dark cycle of 12 h. All experimental procedures were conducted in accordance with the protocol approved by the Ethics Committee for Animal Experiments of Jinan University (Ethics No. 20191021-03). After a one-week acclimatization period, mice were randomly divided into four groups (10 mice in each group), including the normal control (NC) group, the Model (M) group, the leonurine treatment (LH) group, and the fenofibrate treatment (FB) group. Mice in the NC group were given a normal control diet (control feed purchased from Trophic Animal Feed High-Tech Co., Ltd., Nantong, China). Item No.: TP23302, 10% low-fat feed; drinking water: distilled water), while those in the other groups were given a HFHSD (high-fat feed purchased from Trophic Animal Feed High-Tech Co., Ltd., Nantong, China. Item No.: TP23300, 19.4% protein, 20.6% carbohydrate, 60.0% fat; drinking water: high-fructose drinking water: 23.1 g/L fructose + 18.9 g/L sucrose). From the 9th week, the LH group was treated with leonurine by gavage at a dose of 30 mg/kg/d; the FB group was given fenofibrate by gavage at a dose of 40 mg/kg/d, while the other groups were given purified water at the same dosage. During the feeding period, the body weight of mice in each group was tested weekly. At the end of the 12th week, mice were subjected to a 12 h fasting. A small animal anesthesia machine (Ruiwode, R550) was used at an induction concentration of 3% and a maintenance concentration of 1%. Under anesthetized conditions, blood samples were collected from the eye socket vein and the mice were thereby sacrificed. The serum was separated by centrifugation at 1500× *g* for 20 min at 4 °C and stored at −80 °C for biochemical analysis. A portion of liver of each mouse was fixed in 4% paraformaldehyde solution and 2.5% glutaraldehyde electron microscope solution, respectively, for histological analysis, and the remaining liver tissue was then quickly frozen in liquid nitrogen and stored at −80 °C for subsequent analysis.

### 4.3. Histological Analysis

Fresh liver tissue samples fixed with 4% paraformaldehyde were embedded in paraffin and then sliced into a thickness of 4 μm for hematoxylin and eosin (H&E) staining. Additionally, liver tissues fixed with 4% paraformaldehyde were dropped with optimal cutting temperature (OCT) compound embedding agent after dehydration and made into 10 μm frozen slices for oil red O (ORO) staining. An optical microscope (Olympus, Tokyo, Japan) was used to observe H&E-stained sections and ORO-stained sections under 200× fields to detect pathological changes and the accumulation of lipid droplets in liver tissues.

### 4.4. Transmission Electron Microscopy Observations

Fresh liver tissue samples of 1 mm^3^ were fixed in 2.5% glutaraldehyde electron microscope solution at 4 °C for 4 h, washed with phosphate-buffered saline (PBS) 3 times, fixed with 1% OsO_4_ at room temperature for 2 h in the dark, washed with PBS 3 times again, dehydrated, embedded, polymerized, and then cut into ultra-thin sections for staining. The ultrastructure of hepatocytes was observed under a 5800× electron microscope (Thermo Fisher, TECNAIG2 Spirit TWIN, Waltham, MA, USA).

### 4.5. Biochemical Analysis

Hepatic total cholesterol (TC, Cat. A111-1-1, China) and triglyceride (TG, Cat. A110-1-1, China) contents and serum TC, TG, high-density lipoprotein cholesterol (HDL-C, Cat. A112-1-1, China), low-density lipoprotein cholesterol (LDL-C, Cat. A13-1-1, Jiangsu, China), alanine aminotransferase (ALT, Cat. C009-2-1, China), aspartate aminotransferase (AST, Cat. C010-2-1, China), glycated serum protein (GSP, Cat. A037-2-1, China), and glucose (GLU, Cat. A154-2-1, China) contents were analyzed by biochemical kits.

### 4.6. Transcriptomic Analysis

RNA was first extracted and quantified for each sample, and then a high-quality RNA-Seq library was prepared from at least 1 µg of total RNA using the Illumina NEBNext^®^ Ultra^™^ kit, which included steps such as mRNA enrichment, fragmentation, and cDNA synthesis. The resulting libraries were subjected to stringent quality control using a Qubit and Agilent Bioanalyzer. After data quality control and sequence alignment, gene expression was quantified. Differentially expressed genes between the two biological conditions were then identified using DESeq2, which analyzed the unnormalized gene reads’ count data generated by featureCounts. The differential analysis results were adjusted for multiple hypothesis testing using the Benjamani–Hochberg method to control the False Discovery Rate (FDR). Genes were considered differentially expressed if they met the criteria of |log_2_Fold Change (log_2_FC)| ≥ 1 and FDR < 0.05. Following the identification of differentially expressed genes, their functions and interactions were explored, and a gene co-expression network was constructed.

### 4.7. Lipidomic Analysis

Liver samples were taken from a −80 °C freezer and thawed on ice before homogenization at 30 Hz for 20 s with a steel ball, followed by centrifugation at 3000 rpm and 4 °C for 30 s. The homogenized samples (20 mg) were then extracted with 1 mL of a solvent mixture (MTBE–MeOH = 3:1, *v*/*v*) containing an internal standard. After 15 min of vortex mixing, 200 μL of water was added, followed by a 1 min vortex and centrifugation at 12,000 rpm for 10 min. The upper organic layer (200 μL) was collected and evaporated using a vacuum concentrator, and the dry extract was reconstituted in 200 μL of mobile phase B for subsequent LC-MS/MS analysis. Chromatographic separation was performed on a Thermo Accucore^™^ C30 column using a gradient elution with a solvent system consisting of acetonitrile/water (60:40, *v*/*v*) with 0.1% formic acid and 10 mmol/L ammonium formate (solvent A) and acetonitrile/isopropanol (10:90, *v*/*v*) with 0.1% formic acid and 10 mmol/L ammonium formate (solvent B). The flow rate was set at 0.35 mL/min, and the column was maintained at 45 °C. Detection was carried out using an ESI-QTRAP-MS/MS system in both positive and negative ion modes, with source conditions optimized for each ionization mode. MRM transitions were monitored with specific DP and CE values optimized for each target analyte.

### 4.8. Molecular Docking

The crystal structures of ADRA1a, AMPK, and SCD1 used in the docking were downloaded from the AlphaFold database (https://alphafold.ebi.ac.uk/, accessed on 15 January 2024), the 3D structure of Leonurine was downloaded from the PubChem database (https://pubchem.ncbi.nlm.nih.gov/, accessed on 15 January 2024), and energy minimization was performed under the MMFF94 force field. AutoDock Vina 1.1.2 software was used to perform molecular docking [55]. PyMol 2.5.5 was used to remove water molecules, salt ions, and small molecules. The docking box was then set up to encase the entire protein structure. In addition, all processed small molecules and receptor proteins were converted into the PDBQT format required for docking by AutoDock Vina 1.1.2 using ADFRsuite 1.0 [56]. During docking, the elaboration of the global search was set to 32, the output docking conformation with the highest score was considered by us as the binding conformation, and finally the PyMol 2.5.5 docking results were used for visual analysis.

### 4.9. Western Blot

Twenty milligrams of liver tissues from each sample was mixed with 200 μL of radio immunoprecipitation assay (RIPA) lysate buffer (Beyotime, Shanghai, China) containing PMSF (Beyotime, Shanghai, China) and protease inhibitor cocktail (Beyotime, Shanghai, China), homogenized at 50 Hz for 2 min, and then centrifuged at 12,000× *g* for 5 min at 4 °C to extract the protein. The protein contents of the homogenate supernatant were measured using the BCA protein quantitation assay (Beyotime, Shanghai, China), and then the supernatant was mixed with loading buffer and denatured. Five micrograms of protein per sample was loaded onto an 10% sodium dodecyl sulfate-polyacrylamide (SDS-PAGE) gel for electrophoresis and then transferred to PVDF membranes (Millipore, Darmstadt, Germany). Then, 5% skim milk powder was prepared using TBST, blocked for 2 h at room temperature, and membranes were incubated with primary antibodies overnight at 4 °C (1:1000). After being washed with TBST, the membranes were subsequently incubated at room temperature for 1 h with HRP-conjugated secondary antibody (Asbio, 1:2000, Guangzhou, China). Protein bands were covered with chemiluminescent HRP substrates (Millipore, Darmstadt, Germany) and visualized with the ChemiDoc^™^ Touch Imaging System (Bio-Rad ChemiDoc MP, San Francisco, CA, USA). ImageJ was used to analyze the gray values of protein expression.

### 4.10. Immunofluorescence Analyses

The paraffin sections were first treated with water to remove the wax, followed by antigenic repair using microwaves to expose the antigenic sites. Next, the area to be tested was gently circled on the slide to mark the area to be tested, and endogenous peroxidase activity was inactivated with hydrogen peroxide to prevent non-specific reactions. We proceeded to incubate the sections with relevant antibodies, including labeling ADRA1a using fluorescein 488 and repeating the process of antigen repair, primary antibody (1:500), and secondary antibody incubation to label p-AMPKα (1:200). DAPI staining was used to visualize the nuclei, and then the sections were covered with an antifluorescence quenching solution and PVP solution to protect the integrity of the fluorescent signal. Sections were observed with a microscope (Olympus, BX53+DP74) and fluorescent signals were recorded as necessary. At each step, we made sure to rinse the sections with PBS and remove excess reagents. In addition, all fluorescent dye staining should be performed in the dark to maintain the stability of the fluorescent signal. For each experimental condition, three independent experiments were carried out.

### 4.11. Statistical Analysis

Data are expressed as the mean ± standard error of the mean (SEM). GraphPad Prism 9.5 for Windows (GraphPad Software, San Diego, CA, USA) was employed for statistical analyses. Potential significance differences were analyzed by ordinary one-way analysis of variance (ANOVA), and *p* < 0.05 was taken as the statistical significance standard.

## 5. Conclusions

The results show that leonurine changed the gene and lipid profiles of NAFLD in mice, and preliminarily verified that leonurine may have an anti-NAFLD effect through phosphorylation of the ADRA1a/AMPK/SCD1 axis. This conclusion has enriched the understanding of the pathogenesis of NAFLD for drug prevention and NAFLD treatment, alongside other potential targets and pathways. In addition, this study has some limitations, and further verification of the metabolic process of lipid molecules on cell models is needed.

## Figures and Tables

**Table 1:** Table of molecular docking scores for three technological combinations.

Target Name	Ligand Name	Docking Score (kcal/mol)			
		Round 1	Round 2	Round 3	Mean
ADRA1a	Leonurine	−6.751	−6.641	−6.756	−6.716
AMPK	Leonurine	−6.309	−5.613	−6.295	−6.072
SCD1	Leonurine	−8.585	−8.416	−8.473	−8.491

## Data Availability

The data presented in this study are available in the article and Supplementary Materials.

## Supplementary Materials

The following supporting information can be downloaded at https://www.mdpi.com/article/10.3390/ijms251910855/s1.
