# Supplementary material for: Leonurine Inhibits Hepatic Lipid Synthesis to Ameliorate NAFLD via the ADRA1a/AMPK/SCD1 Axis
Source: Int J Mol Sci. 2024 Oct 9;25(19):10855. doi: 10.3390/ijms251910855 (PMC11476755; doi:10.3390/ijms251910855)
Supplement: Supplementary file 1 [file ijms-25-10855-s001.zip › Abbreviations.pdf]

Abbreviations

ACC, acetyl-CoA carboxylase

ADRA1a, alpha-1A adrenergic receptor

ALT, alanine aminotransferase

AMPK, AMP-activated protein kinase

ANOVA, analysis of variance

AST, aspartate aminotransferase

ATP, adenosine triphosphate

cAMP, cyclic adenosine monophosphate

Col3a1, collagen alpha-1(III)

CV, coefficient of variation

DEGs, differentially expressed genes

DG, diglyceride

DGAT, diacylglycerol acyltransferase

FAs, fatty acyls

FASN, fatty acid synthase

FDA, Food and Drug Administration

FPKM, fragments per kilobase of transcript per million fragments mapped

GLs, glycerolipids

GLU, blood glucose

GPs, glycerophospholipids

GSP, glycosylated serum protein

H&E, hematoxylin and eosin

HDL-C, high density lipoprotein cholesterol

HFHS, high-fat high-sugar

HRP, horseradish peroxidase

IL-1β, interleukin-1 beta

KEGG, Kyoto Encyclopedia of Genes and Genomes

LC-MS/MS, liquid chromatography tandem mass spectrometry

LDL-C, low density lipoprotein cholesterol

LH, leonurine treatment group

LPS, lipopolysaccharide

NAFLD, nonalcoholic fatty liver disease

NC, normal control

NF-κB, nuclear factor kappa-light-chain-enhancer of activated B cells

OCT, optimal cutting temperature compound

OPLS-DA, orthogonal partial least squares discriminant analysis

ORO, Oil red O

Osbpl5, oxysterol binding protein like 5

P-AMPKα, Phospho-AMPKα (Thr172)

PBS, phosphate buffered saline

PC, phosphatidyl cholines

PCA, principal component analysis

PE, phosphatidylethanolamines

PLC, phospholipase C

PLC, phospholipase C

PMSF, phenylmethyl sulfonylfluoride

PPAR, peroxisome proliferative activated receptor

PVDF, polyvinylidene Fluoride

QC, quality control

RIPA, radio immunoprecipitation assay

SCD1, stearoyl-CoA desaturase 1

SDS–PAGE, sodium dodecyl sulfate-polyacrylamide

Sms, spermine synthase

SNS, sympathetic nervous system

SEM, standard error of the mean

SREBP-1c, sterol-regulatory element binding protein-1c

TC, total cholesterol

TEM, transmission electron microscopy

TG, triglyceride

TLR4, toll-like receptor 4,

TNF-α, tumor necrosis factor-α

VLDL, very-low-density lipoprotein

WB, western blot
