# Supplementary material for: Leonurine Inhibits Hepatic Lipid Synthesis to Ameliorate NAFLD via the ADRA1a/AMPK/SCD1 Axis
Source: Int J Mol Sci. 2024 Oct 9;25(19):10855. doi: 10.3390/ijms251910855 (PMC11476755; doi:10.3390/ijms251910855)
Supplement: Supplementary file 1 [file ijms-25-10855-s001.zip › FPKM calculation formula & Figure S1.pdf]

FPKM calculation formula

The number of fragments in a transcript is related to the amount of sequencing Data (or Mapped Data), the length of the transcript, and the expression level of the transcript. In order for the fragment number to truly reflect the expression level of the transcript, the number of Mapped Reads in the sample and the length of the transcript need to be normalized. FPKM(Fragments Per Kilobase of transcript per Million fragments mapped) is used as an indicator to measure the level of transcript or gene expression. PKM is calculated as follows:

$$\text{FPKM}=\frac{\text{mapped fragments of transcript}}{\text{Total count of mapped fragments (Millions)} \times \text{Length of transcript (kb)}}$$

mapped fragments of transctipt indicates the number of fragments that are compared to a transcriptome, that is, the number of double-ended Reads, Total Count of mapped fragments(Millions) indicates the total number of fragments that are compared to a transcriptome, In units of 10^6; Length of transcript (kb): Length of transcript, in units of 10^3 bases.

1.1. Figure S1

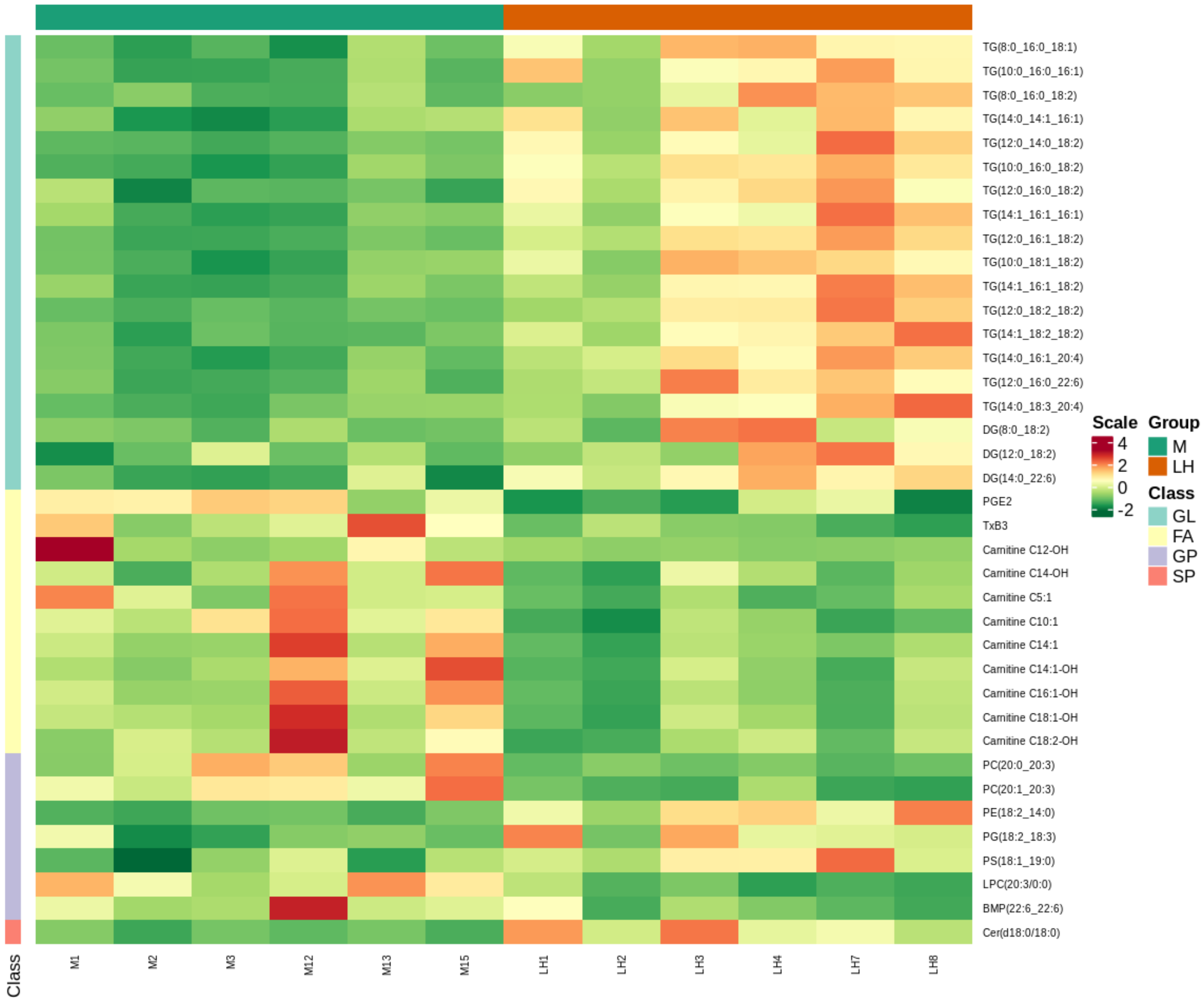

Figure S1. Cluster heatmap analysis of differential lipid molecules in the M and LH groups.
